# Study protocol: Imaging brain development in the Childhood to Adolescence Transition Study (iCATS)

**DOI:** 10.1186/1471-2431-14-115

**Published:** 2014-04-30

**Authors:** Julian G Simmons, Sarah L Whittle, George C Patton, Paul Dudgeon, Craig Olsson, Michelle L Byrne, Lisa K Mundy, Marc L Seal, Nicholas B Allen

**Affiliations:** 1 Melbourne School of Psychological Sciences, The University of Melbourne, VIC 3010, Australia; 2 Murdoch Childrens Research Institute, Melbourne, Australia; 3 Melbourne Neuropsychiatry Centre, Department of Psychiatry, The University of Melbourne, 1-100 Grattan Street, Parkville, VIC 3010, Australia; 4 Centre for Adolescent Health, The Royal Children’s Hospital, Melbourne, Australia; 5 Deakin University, Melbourne, Australia; 6 Department of Paediatrics, The University of Melbourne, Melbourne, Australia

**Keywords:** Puberty, Hormones, Adrenarche, Adolescence, Brain development, Protocol, MRI, Gonadarche

## Abstract

**Background:**

Puberty is a critical developmental phase in physical, reproductive and socio-emotional maturation that is associated with the period of peak onset for psychopathology. Puberty also drives significant changes in brain development and function. Research to date has focused on gonadarche, driven by the hypothalamic-pituitary-gonadal axis, and yet increasing evidence suggests that the earlier pubertal stage of adrenarche, driven by the hypothalamic-pituitary-adrenal axis, may play a critical role in both brain development and increased risk for disorder. We have established a unique cohort of children who differ in their exposure to adrenarcheal hormones. This presents a unique opportunity to examine the influence of adrenarcheal timing on brain structural and functional development, and subsequent health outcomes. The primary objective of the study is to explore the hypothesis that patterns of structural and functional brain development will mediate the relationship between adrenarcheal timing and indices of affect, self-regulation, and mental health symptoms collected across time (and therefore years of development).

**Methods/Design:**

Children were recruited based upon earlier or later timing of adrenarche, from a larger cohort, with 128 children (68 female; M age 9.51 years) and one of their parents taking part. Children completed brain MRI structural and functional sequences, provided saliva samples for adrenarcheal hormones and immune biomarkers, hair for long-term cortisol levels, and completed questionnaires, anthropometric measures and an IQ test. Parents completed questionnaires reporting on child behaviour, development, health, traumatic events, and parental report of family environment and parenting style.

**Discussion:**

This study, by examining the neurobiological and behavioural consequences of relatively early and late exposure to adrenarche, has the potential to significantly impact our understanding of pubertal risk processes.

## Background

The transition from childhood to adolescence is a period of opportunities (as young people physically and sexually mature, and peer and familial relationships change), challenges (as responsibilities and exposure to a range of risks, such as substance use and sexual activity, increase), and vulnerabilities (with half of all lifetime cases of mental illness starting by age 14 years) [1]. The transition coincides with the biological processes of puberty and changes in brain structure and function [2,3]. Indeed, it is increasingly being recognised that pubertal maturation may influence brain development, and in turn, psychosocial maturation. In this regard, the *timing* of pubertal stage relative to peers is relevant. Early pubertal timing predicts adolescent onset mental illness and other poor outcomes during adolescence, and in some cases better accounts for sex differences in the onset of psychopathology than age [4,5]. Specifically, early pubertal timing has been consistently associated with depressive symptoms in girls [6,7], while findings for boys are mixed [8]. Further, advancing pubertal stage increases risk for both the onset and persistence of depressive symptoms in girls [9] and violent and anti-social behaviours in both sexes [10].

The current literature on puberty and brain development has many general methodological limitations [see [11], and specifically has not addressed the differential effects of adrenarche (the earlier phase of pubertal development driven by the hypothalamic-pituitary-adrenal [HPA] axis) and gonadarche (the later phase driven by the hypothalamic-pituitary-gonadal [HPG] axis). Despite the fact that the timing of both phases of pubertal development have been associated with behavioural and mental health problems [6,7,12], most research has focused on gonadarche and little is known about the earlier adrenal phase of pubertal development. This emphasis has likely been a product of the relative difficulty in assessing adrenarcheal development due to the paucity of early physical signs, and that adrenarche has only been detected in humans and some higher primates [13], limiting experimental animal work.

This paper is a methodological description of the *Imaging brain development in the Childhood to Adolescence Transition Study* (iCATS), which was embedded in a larger *Childhood to Adolescence Transition Study* (CATS) [14]. Both of these studies aim to address existing gaps in knowledge. However, iCATS will specifically, and uniquely, examine the influence of adrenarcheal timing on brain development and function, and on mental health.

### Puberty and adolescent brain development

The principal focus in studies of adolescent brain development was traditionally on age-related changes [e.g., [15]. Based on findings (e.g., peaks in grey matter density that appear to coincide with timing of puberty onset, [16]) researchers have more recently suggested that brain development patterns align better with pubertal changes rather than with age. It is well established that steroid hormones play critical roles in development [17]. However, the extant literature remains sparse, and predominantly focuses on gonadarcheal indices. Importantly, although there is informative animal work regarding the links between gonadarcheal hormones and brain structure and function, relatively little is known about this relationship in humans [16] and particularly in relation to adrenarcheal hormones [18].

Recently, a small number of adolescent MRI studies have investigated, in more detail, the relationships among brain structure and function, gender, and hormones that change at puberty. A structural MRI study by Peper and colleagues [19] with 10–15 year olds showed an association between testosterone levels and global grey matter density in males (and not in females). Neufang and colleagues [20] examined 46 participants aged 8–15 years and found a positive relationship between Tanner stage and testosterone levels and grey matter volume in the amygdala, and a negative relationship between these measures and hippocampal volume, regardless of gender. In addition, males showed a negative relationship between testosterone and parietal cortex grey matter. In a more recent study specifically examining adrenarcheal hormones, Nguyen et al. [21] explored associations between androgens and cortical thickness and found that DHEA levels were positively associated with cortical thickness in various frontal, parietal, and temporal cortical regions from various ages between 4–13 years. For instance, DHEA was positively associated with cortical thickness of the left dorsolateral prefrontal cortex from ages 4 to 8 for males and females. Conversely, sex-specific changes were observed in the association between testosterone and cortical thickness. Specifically, pre-pubertal males (defined as Tanner Stage 1 or 2) typically demonstrated negative associations between testosterone and cortical thickness, whereas pre-pubertal females demonstrated positive associations. Trajectories of white matter development have also been found to differ as a function of pubertal hormones. Peper and colleagues (2009) reported a positive association between gonadotropic hormones and white matter density at age nine. A study by Perrin and colleagues [22] found that trajectories of white matter development in males were related to expression levels of a gene encoding a testosterone receptor, suggesting that effects of testosterone may be responsible for the sexually dimorphic relationship between age and white matter volume. These studies demonstrate promising evidence that pubertal hormones influence structural brain development in humans.

Few studies have investigated the effects of puberty on brain function in normal samples. Forbes and colleagues [23] found that those with more advanced pubertal maturation (measured by Tanner stage) exhibited less striatal and more medial prefrontal reactivity to reward, and that testosterone was positively correlated with striatal reactivity in boys during reward anticipation and negatively correlated with striatal reactivity in girls and boys during reward outcome.

Importantly, these published studies have not investigated possible effects of the timing of pubertal events on brain development, highlighted as a critical area for future investigation [16]. We have recently completed the first study of the relationship between pubertal timing, brain structure and depressive symptoms during early adolescence [24]. We found that larger volume of the pituitary gland, a key component of the HPG and HPA axes, mediated the relationship between early pubertal timing and depressive symptoms in 155 adolescents (72 females). Relatedly, we also found that pituitary volume in early adolescence predicts HPA activity in mid-adolescence in the same cohort [25]. These findings are consistent with neurobiological mechanisms being responsible for the link between early pubertal timing and depressive symptoms in adolescents.

However, as these studies only used self- and parent-report measures of pubertal development and did not differentiate adrenarche and gonadarche, they cannot differentiate the relationship between specific adrenarcheal hormones, brain development and function. Furthermore, the focus on one brain region (the pituitary gland), although important, did not allow investigation of the role of brain development more broadly in these processes.

### Imaging brain development in the Childhood to Adolescence Transition Study (iCATS)

Funding was obtained from the Australian Research Council (ARC; DP120101402, 2012 – 2014) to recruit two groups of participants stratified by hormonal indices of pubertal timing, selected from the larger population-based CATS investigation (N = 1239 Grade 3 [667 females; 7.79 – 10.65 years of age]) [14]. This permitted the investigation of the associations between the timing of pubertal maturation (as assessed by gender-specific hormonal and body morphological changes) during early pubertal development, namely adrenarche (funded) and gonadarche (to be funded), on aspects of brain structure and function implicated in emotional dysregulation and mental disorder.

### Aims

The broad aim of iCATS is to elucidate which children are most at risk for adverse outcomes as they pass through puberty. The specific aim of iCATS is to identify the neurobiological mechanisms that mediate this risk. The primary objective of this study is to explore the hypothesis that patterns of structural and functional brain development will mediate the relationship between pubertal timing and the indices of affect, self-regulation, and mental health symptoms collected across waves (and therefore years of development).

Current understanding of the relationship between puberty and brain development is rudimentary, with few studies looking specifically at the effects of pubertal timing [24]. We hypothesise, based on extant literature, that earlier exposure to adrenarche will be associated with neurodevelopmental and behavioral outcomes that have been previously associated with greater risk for mental health problems. Our specific aims are to:

1. Investigate the relationship between adrenarcheal timing and brain structure in both sexes, by examining associations between advanced adrenarcheal development, measured by DHEA, DHEA-S and testosterone, and (i) cortical grey matter, (ii) white matter, (iii) limbic, and (iv) striatal volumes.

2. Investigate the relationship between adrenarcheal timing and brain function (both at rest and in the context of affective processing) in both sexes, by examining associations between advanced adrenarcheal development and both function and connectivity in (i) limbic, (ii) prefrontal, and (iii) parietal regions.

3. Investigate if (i) stressful life events, (ii) long-term cortisol levels and (iii) immune system function moderate the associations between adrenarche and brain structure and function described in Aims 1 and 2.

4. Investigate the relationship between Aims 1 – 3, health and risk, by examining whether the patterns of structural and functional brain changes identified above mediate the relationship between pubertal development and indices of affect, self-regulation, and mental health symptoms.

## Methods/Design

### Design

The funded component of iCATS is a cross-sectional study of the relationship between pubertal timing and brain structure and function in children approximately 9 years old. At this time the processes of adrenarche are at the fore. Children were selected and invited to take part in iCATS based upon hormone levels collected at a baseline assessment from the broader CATS [14] (M age = 8.98, SD = 0.39 years). Children were grouped as either relatively early adrenarche or late adrenarche, based upon the entire distribution (see Selection Strategy below). Children took part in iCATS an average of 27.78 weeks (SD = 8.45) after their CATS participation. iCATS is based in the Melbourne School of Psychological Sciences at the University of Melbourne, Australia. Ethics approval was granted by the Royal Children’s Hospital Human Research Ethics Committee (#32171), and ratified by the University of Melbourne Human Research Ethics Office (#1238745).

Further funding will be sought to enable the current investigation to be repeated in later years, allowing examination of latter adrenarcheal and gonadarcheal processes, as well as longitudinal and prospective relationships with brain development and function and health outcomes.

### Recruitment

Recruitment was restricted to the CATS cohort [see [14], and only to those families with active consent who had taken part in all CATS baseline assessments. The parents of children selected for iCATS were sent participant information and consent (PICF) documents by post, and followed up with a phone call at least seven days later. Parents were given the option to decline contact (and thus participation) via email, phone or mobile phone text message. School principals were notified and informed about the study two weeks prior to PICF documents being sent to families, and teachers one week prior. Children attending schools and/or with parents who were identified by CATS staff as reticent about participation were not invited to take part in iCATS (N = 94 of total CATS cohort). However, their hormonal data was left in the total distribution calculations (see below).

### Selection strategy

Participants were characterised, based upon stratification of their relative pubertal development in the CATS baseline assessment, into two groups: (1) a relatively early adrenarcheal group, and (2) a relatively late adrenarcheal group. Measuring pubertal development, i.e. progress in adrenarche, in children of this age remains an area of conjecture; particularly as few, if any, physical signs of development are detectable [11,26]. Therefore, as the primary aim of iCATS was to examine the effect of adrenarche on brain development, and the hypothesised mechanism of action is via hormones, adrenarcheal hormones (i.e. DHEA, DHEA-S, and testosterone) were assessed in order to inform the selection of groups. There was a high degree of correlation between DHEA and testosterone in the larger cohort (males: *r* = .69, *p* = .000; females: *r* = .65, *p* = .000), and previous work has established that DHEA and testosterone levels show strong correspondence with physical examinations and a picture based interview at Tanner stages I – V [26].

Although testosterone is typically associated with gonadarche, it is also metabolized in the adrenal zona reticularis and peripheral tissue after the conversion of DHEA to androstenedione [27,28]. Notably, these non-gonadal pathways are the primary source of testosterone in males and females pre-gonadarche, and females post gonadarche. Therefore, children with relatively advanced adrenarche will demonstrate high DHEA and testosterone levels, and those with relatively delayed adrenarche will show low levels of DHEA and testosterone. Adrenarche was therefore modeled here by plotting DHEA and testosterone hormone levels from the baseline CATS saliva collection, with the crossover area of the upper tertiles of DHEA/testosterone characterised as relatively earlier development (ED), and the lower tertiles as relatively later development (LD). Group allocation will be re-examined with the iCATS hormone and parent report data collected (see below). Inclusions applied prior to this modeling were an age between 8 and 10 years, and a BMI between the 5^th^ and 95^th^ percentiles of standardised growth for children and adolescents [29].

### Participants

Participants comprise 128 children (*M* age = 9.51 years, SD = 0.36) and a parent/guardian (84% were mothers), with 66 children (35 female) participating from the ED group, and 62 (33 female) from the LD group. A total of 377 families were invited to take part, however 241 declined to participate and a further 8 were excluded based upon eligibility criteria (see Table 1).

**Table 1:** Eligibility criteria for iCATS

**Inclusion Criteria**	**Exclusion Criteria**
Parental and child consent in CATS;	History of head trauma or loss of consciousness;
Completed Wave 1 of CATS in full, i.e., parent/guardian q’s, and child q’s, anthropometric measurements, and saliva sample;	History of clinically significant developmental or intellectual disorder;
Child aged between 8.5 and 9.5 years at the time of their CATSparticipation;	Clinically significant DHEA, DHEA-S, Testosterone;
Written consent provided by parent for their own participation;	Indications of claustrophobia;
Written consent provided by the parent and the child for the child’s participation; and,	Long-term use of steroidal or amphetamine based medications;
Verbal consent provided by the child.	Short-term current use (i.e., < 2 weeks) of amphetamines;
	Short-term current use of steroidal medications will be reviewed on a case-by-case basis;
	Presence or likelihood of internal or external non-removable ferrous metals;
	Inability or unwillingness of participant or parent/guardian to provide informed consent.

### Data collection

Participating families were visited at home, and then asked to attend a session at The Royal Children’s Hospital (RCH) in Melbourne, Australia.

#### Home visit

The home visit initially comprised a review of study participation requirements, eligibility, PICF documents, and MRI familiarization video, as well as questions from the family. Families were informed that participation in iCATS had no bearing on participation in the larger study, and that they were free to complete some components and not others. Families were advised that all their information is confidential, except where limited by law, and that information collected will not be fed back to them, except where clinically significant abnormalities were indicated. Signed consent from a parent/guardian and verbal consent from children was required.

Measures collected during the home visit included demographic and MRI safety information, anthropometric measures, parent questionnaires, and the collection of a hair sample from the child. Further, the procedure for the collection of the saliva samples on the day of, and day prior to, the RCH visit were explained and demonstrated.

#### RCH Visit

Visit requirements and consent was reviewed, and children asked to complete questionnaires. Research staff reviewed children’s responses to questionnaires and any indications of risk or clinically significant pathology were followed up with investigators (at the time or later, dependent on level of risk), and parents and children as appropriate. Children and parents were then run through an MRI scanner familiarisation session, to decrease anxiety, increase participation rates and compliance/stillness during the scans. All research staff administering the familiarisation sessions were required to undergo training at RCH through the Developmental Imaging Group of MCRI.

An MRI technician at RCH verbally reviewed the MRI safety checklist with parents and children just prior to undertaking the MRI session, and children were asked to choose a cartoon or movie they would like to watch during the scans (excluding the fMRI sequences). Parents were asked to remain in the MRI room while scanning was carried out. Subsequently, children were positioned comfortably in a supine orientation with their head located in a head-RF coil that was electrically isolated. The participant viewed a screen, via an angled adjustable mirror, on which all visual stimuli or video were presented using a back-projection system attached to a computer. Children wore MR-compatible headphones to reduce MRI noise, to allow them to hear instructions and speak with the MRI technician, and to hear the audio of any cartoons or movies they watched. Children were provided with an “Emergency Stop” button, in order to indicate to research staff if at any stage during the scan they felt distress and wanted to cease the procedure. Children completed a T1-weighted MPRAGE structural sequence, followed by two fMRI sequences (rest [eyes closed], and an affective faces task), and finally a diffusion weighted imaging sequence.

In cases where technical error or movement required a particular sequence be repeated, a case-by-case assessment was made by research staff in discussion with the parent, child and MRI technician. Scanning took an average of 45 minutes.

After the scan families were given a break and then children completed the intelligence quotient (IQ) test. Finally, parents and children took part in a debriefing interview.

### Measures

#### Demographics and health information

Detailed demographic information was collected as part of the larger study [14], including family composition, parental education and age, annual household income, language spoken at home, ethnicity and adoption, and this data will be available for iCATS analyses. However, critical information was checked with parent and child, including names, dates of birth, and contact details. Questions were also asked covering health information related to eligibility criteria and MRI safety exclusions, as well as illnesses and stressful events experienced in the prior three months.

#### Magnetic resonance imaging

Neuroimaging data were acquired on the 3T Siemens TIM Trio scanner (Siemens, Erlangen, Germany) at the MCRI, RCH, Melbourne. Participants lay supine with their head supported in a 32-channel head coil.

##### Structural scan

T1-weighted images were acquired during a 3.5 minute sequence (repetition time = 1900 msec; echo time = 2.24 msec; flip angle = 9°, field of view = 23 cm^2^), which produced 176 contiguous 0.9 mm thick slices (voxel dimensions = 0.9 mm^3^).

##### DWI

Two diffusion weighted sequences were included in the protocol. The first sequence was optimised for generation for diffusivity maps (60 directions; b = 1000 s/mm^2^, repetition time = 8800 msec; echo time = 99 msec; slices = 64; voxels = 2 x 2 x 2). The second diffusion sequence was optimised for tractography (HARDI: 67 directions; b = 3000 s/mm^2;^ repetition time = 8100 msec; echo time = 113 msec; slices = 54, voxels = 2.3 x 2.3 x 2.3).

##### Resting fMRI

A single 6-minute continuous functional gradient-recalled acquisition sequence was conducted at rest to acquire 154 whole-brain T2*-weighted echo-planar images (repetition time = 2400 ms, echo time = 35 ms, pulse angle = 90°) within a field of view of 126 mm, with a voxel size of 3.3 × 3.3 × 3.3 mm. Thirty-eight interleaved slices were acquired. Complex field maps were obtained in order to correct for distortion caused by magnetic field inhomogeneities.

##### Passive face viewing fMRI task

Participants were administered a modified version of a common face emotion-viewing task used previously with children [30]. As in these prior studies, children were presented with a series of faces varying in affective content and asked to complete a simple button press each time a face appeared (to ensure maintenance of attention). A less constrained response than other typical affective face paradigms was chosen given the young age of our child participants. Calm, happy, angry, and fearful facial expressions from the Nimstim Set of Facial Expressions (NimStim; http://www.macbrain.org/resources.htm) were presented in two 3 minute 40 second runs using a block design. Each run contained 1 block of each face type, with each containing 8 faces (randomly presented, 4 male and 4 female) presented for 3 seconds each, separated by a 1 second fixation. Blocks were separated by 15 second fixation rests. Given the high percentage of Caucasian participants, and the predominant use of Caucasian faces in Australian face-processing research, only Caucasian faces were used in the paradigm. Block order was counterbalanced across participants. During each run, 70 whole-brain T2*-weighted echo-planar images (repetition time = 3000 ms, echo time = 40 ms, pulse angle = 85°) within a field of view of 120 mm, with a voxel size of 3 × 3 × 3 mm. Forty interleaved slices were acquired.

#### Saliva samples

Children, with the help of a parent/guardian, were asked to collect a saliva sample on the day of and day prior to their RCH visit immediately after waking, and prior to the consumption of food or tooth brushing. This was collected via the passive drool of whole saliva using a straw into test tubes (all equipment provided). Families were given a stopwatch to allow them to record how long it took the child to provide enough saliva to reach the marked 2.5 ml line on the tube. Samples were then frozen in family’s freezers in provided sealed containers, and subsequently transported in provided coolers packed in Techni-Ice^TM^ to RCH on the day of the MRI visit. Families were asked to minimize the time the samples spent out of the freezer, and no samples were found to have risen above 0°C upon arrival at RCH. Samples were then assayed an average of 7.72 weeks (SD = 4.84) after collection, with one freeze/thaw cycle. At time of assay, samples were defrosted and centrifuged, with the supernatant assayed for levels of testosterone, DHEA and DHEA-S, as hormonal markers of adrenarcheal development. Remaining supernatant was stored in 1 ml aliquots in a -80C freezer for future assays. An aliquot was subsequently assayed for immune system biomarkers, including C-reactive protein (CRP) and secretory immunoglobulin A (SIgA). Salivary assays of each of these biomarkers are now well-accepted substitutes for measuring serum levels [31,32], although there are methodological idiosyncrasies for each. For example, DHEA-S must pass between cells to be excreted in saliva and thus salivary flow rate is an important determinant of the index. Hormonal assays were conducted at MCRI, using Salimetrics ELISA kits. Kits from the same lot numbers were used, as were in-house controls. The inter-assay coefficients of variation (CVs) were: DHEA = 5.45%, DHEA-S = 7.53%, testosterone = 13.54%. The intra-assay CVs were: DHEA = 8.56%, DHEA-S = 9.38%, testosterone = 7.32%.

#### Hair sample

Hair samples were collected for the assay of long term cortisol levels. Cortisol levels are most commonly assessed via saliva, urine and serum collections. However, variable findings in the cortisol/stress literature may be due to methodological differences in the timing, number and type of cortisol measures taken, likely due to circadian cycling, and high intra-individual variability and reactivity. Hair, a relatively new measure of cortisol, which although unable to demonstrate HPA reactivity temporally linked to a short term stressor, is able to provide a reliable indicator of long term HPA function [in the order of months, [33]. Hair grows at approximately 1 cm per month, thus 3 cm lengths of hair will provide an index of cortisol over the preceding three months. Samples were collected from an area approximately 1 cm^2^ on the posterior vertex of the scalp, as this has proven the most reliable area for stable cortisol levels. Hair samples were not collected where <3 cm of hair length was available, to minimize cosmetic impact, therefore significantly fewer boys provided these samples. Hair cortisol assays were conducted by Stratech Scientific, where samples were cut down to 3 cm lengths, and processed and assayed as described previously [34], using Salimetrics ELISA cortisol kits. The inter-assay coefficient of variation (CV) was 6.7%, and intra-assay CV 4.9%.

#### Anthropometry

Two measurements were obtained for height, weight and waist circumference. A third measurement was obtained where the prior two were not within a specified range (0.5 cm for height, 0.1 kg for weight, 0.5 cm for waist). The mean value was used in any further calculations if two measurements were taken, and the median value was used if three measurements were obtained.

##### Height

Was measured to the nearest 0.1 cm using a portable rigid Invicta stadiometer. Height was transformed to z-scores based upon age and gender related reference charts [35].

##### Weight

Was measured to the nearest 0.1 kg using calibrated Tanita THD 382 digital scales. Children were asked to remove shoes, coats and heavy clothing items. Body Mass Index (kg/m^2^) was calculated and transformed, along with student weight, to z-scores based upon age and gender related reference charts [35].

##### Waist circumference

Was measured with a non-stretch anthropometric tape according to the International Society for the Advancement of Kinanthropometry (ISAK) protocols. Waist circumference is an acceptable, non-invasive and reliable indicator of intra-abdominal fat and was measured to the nearest 0.1 cm [36].

#### Parent questionnaires

Questionnaires completed by parents/guardians comprised reports on child, and self-report. The broad domains questionnaires fall into are child pubertal development, internalising-externalising behaviour, and traumatic events, as well as, parental report on the family environment and parenting style.

##### The Pubertal Development Scale

Parent Report (PDS-PR) is a non-invasive questionnaire measure of pubertal development, and is based on the scale of Petersen and colleagues [37]. The version used is completed by parents, and for female children comprises nine items (including date of onset for menarche, if applicable), and for male children comprises 11 items. The PDS-PR has been demonstrated to be both a valid and reliable measure of pubertal development in children, showing high levels of consistency with other more direct measures of development [26,38].

##### The Sexual Maturity Status

Parent Report (SMS-PR) is a series of stylised line drawings of girls/boys bodies at differing stages of pubertal development [39]. Parents with a female child are asked to look at a page with five stages of breast development, and five stages of hip and pubic hair development, and asked to circle a number above the two images that most accurately represent their daughter’s development. Parents with a male child complete the same task, but with five stages of male genital and pubic hair development in the one set of five images. These images directly correspond to the Tanner stages of pubertal development, and have shown good reliability with physician ratings [11].

##### The Child Behaviour Checklist

Parent Report (CBCL-PR) is a parent/guardian questionnaire in which a child's problem behaviours and competencies are rated, with a focus on internalising and externalising behaviours [40]. The age range for children is 6 – 18 years and it provides empirically based syndromes scales scored from factor analyses including Anxious/Depressed, Withdrawn/Depressed, Somatic Complaints, Social Problems, Thought Problems, Attention Problems, Rule-Breaking Behaviour, and Aggressive Behaviour. The CBCL also provides DSM-IV scales, including Affective Problems, Anxiety Problems, Somatic Problems, Attention Deficit/Hyperactivity Problems, Oppositional Defiant Problems, and Conduct Problems. The CBCL has a comprehensive normative sample across ethnicities and SES [40].

##### The Lifetime Incidence of Traumatic Events

Parent Report (LITE-PR) is a checklist covering aspects of a child’s history of potentially traumatic events. The checklist, devised by Greenwald and Rubin [41], allows parents to rate which types of traumas or losses their child has experienced, at what age, how many times, how upset the child was at that time, and how much it bothers them currently. Items can be classified into categories: accidents, witnessing an injury to a loved one, bereavement, natural disasters, abuse (both physical, sexual and emotional), witnessing domestic violence and being a victim of crime. The 16 item LITE was adapted here, by removing the items on sexual assault, and adding two new items covering mother-child separations and domestic relocation. The LITE was designed as a screener, and thus no agreed scoring system has been developed. For the purposes of the present investigation each item will be tallied to give a score for the total number of types of trauma, and another score for the overall number of traumas experienced. The level of upset/bother caused by each type of trauma indicated will also be scored (e.g., 0 = none, 1 = some, 2 = lots) and tallied to give a score for the level of emotional upset experienced when the trauma occurred, and how much it bothers them now.

##### The Alabama Parenting Questionnaire (APQ)

Measures five dimensions of parenting that are relevant to the aetiology and treatment of child externalising and internalising problems: (1) positive involvement with children, (2) supervision and monitoring, (3) use of positive discipline techniques, (4) consistency in the use of such discipline and (5) use of corporal punishment [42]. Factor analyses have substantiated the five factor subscales [43], and the questionnaire has been utilised and published widely with a range of populations. The APQ has normative data in the relevant age range, as well good evidence for reliability and validity [44].

##### The Multidimensional Neglectful Behaviour Scale

Parent Report (MNBS-PR) was developed to estimate the prevalence of neglect and specific sub-types of neglect in community samples, with differing versions for different age probands [45,46]. The MNBS for 5 – 9 year olds was used to measure the extent to which the following needs of children are or have been neglected: physical needs such as food, clothing, shelter, medical care; emotional needs such as affection, companionship, support; supervision needs such as limit setting, attending to misbehaviour, knowing child's whereabouts and friends; and, cognitive needs such as being played with or read to, assisting with school homework versions measure the following four aspects of neglect. Parents were asked to consider the past six months and report on each item. Adequate levels of reliability and validity have been reported [46].

#### Child questionnaires

##### The Children’s Depression Inventory 2 (CDI-2)

Is a brief self-report test that helps assess cognitive, affective and behavioural signs of depression in children and adolescents 7 to 17 years old. The CDI-2 has two scales (Emotional Problems, Functional Problems) and four subscales (Negative Mood/Physical Symptoms, Negative Self-Esteem, Interpersonal Problems, Ineffectiveness). The CDI-2 has normative data in the relevant age range, as well solid evidence for reliability and validity [47].

##### The Spence Children’s Anxiety Scale (SCAS)

Is a brief self-report test of anxiety symptoms broadly in line with the dimensions of anxiety disorder proposed by the DSM-IV. The scale assesses six domains of anxiety including generalized anxiety, panic/agoraphobia, social phobia, separation anxiety, obsessive-compulsive disorder and physical injury fears. The SCAS has normative data in the relevant age range, as well solid evidence for reliability and validity [48,49].

##### Positive Affect and Negative Affect Schedule

Child Form (PANAS-C) is a self-report questionnaire used to measure the respondent’s emotions. The state and trait forms of the questionnaire was used, indexing emotions for “right now” and during “the past few weeks”, respectively. It lists single commonly used adjectives (e.g., *Interested, Sad, Frightened, Alert*), and items are grouped into subscales: positive affect and negative affect. The respondent is asked to read words that describe feelings and emotions and enter a number that corresponds to the value on a scale. The 5-item scale ranges from “not at all”, with a value of 1, to “a lot” with a value of 5. The PANAS-C has been used successfully on large studies of children and exhibits high reliability and good convergent and discriminant validity [50].

##### Edinburgh Handedness Inventory (EHI)

Can be administered as an interview, observational assessment or self-report questionnaire to establish the dominant hand [51]. Items consist of a series of activities, and participants are asked to indicate their hand preference for each. The EHI is completed in the current study as an observational interview, where children are asked to mimic the activity described. This decreases self-report perceived dominant hand bias. The handedness preference score is used in the analysis and interpretation of MRI data and the examination of laterality effects.

#### Intelligence quotient test

The Wechsler Abbreviated Scale of Intelligence (WASI) is a standardized brief measure of general intelligence. Children completed the two-subtest form, comprising Vocabulary and Matrix Reasoning, which provides an estimate of Full Scale IQ (FSIQ) and only takes 15 minutes to complete. Norms are based on a national U.S.A. cohort of 2300 participants between the ages of 6 and 90 years.

### Sample size

Using G*Power 3.1.5 [52], power analyses reveal that 120 participants will be sufficient to achieve power equal to at least 80% for standardized moderating effects of at least 0.26 between continuous variables (equivalent to an improvement in R-squared for the moderating term of 0.07) when α = .05. The same sample size is sufficient to achieve power equal to at least 80% for moderate or larger mediating effects (i.e., a standardized effect of about 0.39) where the independent variable to mediator, and mediator to dependent variable relations are of medium sizes (i.e., standardized regression paths of 0.26 − 0.39) [53]. Results reported in analogous published studies of brain structure and functioning in pubertal development have all reported much larger effect sizes with smaller sample sizes [e.g., 23, 24], thereby indicating that the current study has appropriate statistical power given current standing of knowledge in this area.

### Data analysis

The primary method of analysis for all four aims of the study will be multiple linear regression. The dependent variable for aims 1 – 3 will be the relevant brain structure measure or activation measure referred to in the hypothesis. The dependent variables for aim 4 will be the collected indices of affect, self-regulation, and mental health symptoms. Both whole brain and region of interest (ROI) analyses will be conducted using FreeSurfer and SMP8 software for brain grey matter structure and function, respectively. For aims 3–4, structural and functional measures will be extracted from ROIs defined either anatomically or based on the findings from aims 1–2. The MRtrix package (JRD Tournier, Brain Research Institute, Melbourne, Australia, http://www.brain.org.au/software/) will be used to perform white matter tractography with diffusion weighted MRI, resolving crossing fibres using Constrained Spherical Deconvolution [54]. Hypotheses 3 – 4 will be investigated using linear regression models and bias-estimated bootstrapping techniques to test for moderated mediation.

## Discussion

There are good reasons to suspect that pubertal hormonal changes affecting cognitive and affective systems in the brain, and the resultant psychological changes, most powerfully explain the emergence of symptoms of mental disorder. However the extant human literature on the relationship between pubertal processes and brain development is preliminary at best, and often confounds aspects of pubertal development such as stage, timing and chronological age. Furthermore, although adrenarche and gonadarche are clearly distinct phases of pubertal development, with differing underlying biological mechanisms, their differential impacts on brain development have not yet been examined in a comprehensive study. Compared to other phases of development, puberty and its disorders remain unexplored and poorly understood. This study, by examining the neurobiological and behavioural consequences of relatively early and late exposure to adrenarche, has the potential to profoundly change our understanding of pubertal risk processes. Work on preventive interventions suggests the feasibility of intervening in the social contexts of younger adolescents, but the further development of such interventions is now limited by our understanding of how puberty interacts with the social context and lifestyles of young people to generate health problems.

## Abbreviations

APQ: The alabama parenting questionnaire; BMI: Body mass index; CBCL: Child behaviour checklist; CDI-2: Children’s depression inventory version 2; DHEA: Dehydroepiandrosterone; DHEA-S: Dehydroepiandrosterone sulphate; EHI: Edinburgh handedness inventory; HPA: Hypothalamic-pituitary-adrenal axis; HPG: Hypothalamic-pituitary-gonadal axis; iCATS: Imaging brain development in the childhood to adolescence transitions study; ISAK: International society for the advancement of kinanthropometry; LITE: Lifetime incidence of traumatic events; MRI: Magnetic resonance imaging; MNBS-PR: The multidimensional neglectful behaviour scale – parent report; PDS: Pubertal development scale; PANAS-C: Positive affect and negative affect schedule — child form; RA: Research assistant; SCAS: Spence children’s anxiety scale; TEM: Technical error of measurement.

## Competing interests

The authors declare that they have no competing interests.

## Authors’ contributions

GP, NA, SW, CO and JS contributed to the overall design and conception of the study and assisted with the writing of the grant application. JS, SW and NA drafted and revised this manuscript. JS, SW, MB, LM, GP, MLS and NA contributed to study implementation and coordination. PD contributed to the statistical design of the study. All authors read and approved the final manuscript.

## Pre-publication history

The pre-publication history for this paper can be accessed here:

http://www.biomedcentral.com/1471-2431/14/115/prepub
